# Pre-service teachers’ altruistic motivation for choosing teaching as a career: where does it come from?

**DOI:** 10.3389/fpsyg.2024.1334470

**Published:** 2024-04-02

**Authors:** Zhuotong Li, Wu Yuan Guo

**Affiliations:** ^1^School of Philosophy and Social Development, South China Normal University, Guangzhou, China; ^2^Haile Experimental School, Shenzhen, China

**Keywords:** pre-service teachers, altruistic motivation, process-motivation theory, career choice, qualitative study

## Abstract

This study aims to examine the factors influencing pre-service teachers’ altruistic motivation for selecting their profession within a Chinese educational setting. Drawing on existing research, a three-element (social-cognitive, emotional, and realistic) model is integrated to explore how pre-service teachers’ altruistic motivations are formed and evolved. Utilizing this model, interview data from 18 students enrolled in the Chinese Free Teacher Education program were collected and analyzed by thematic analysis. The findings indicate that social-cognitive factors impact altruistic motivation through engagement with social issues and reflections on practical educational challenges. The emotional factor is manifested through the participants’ positive and negative emotions. The realistic factor comprises familial influences and personal career preferences, which play a role in the decision to pursue teaching as a lifelong vocation. This study proposes a structured and functional model that can serve as a foundation for future research into the development of altruistic motivation. It also offers insights into nurturing altruistic motivation among both pre-service and practicing teachers in their career decision-making process.

## 1 Introduction

Teaching is often regarded as one of the most significant and respected professions in contemporary society (Wilkins and Comber, 2015). However, teachers are currently faced with numerous career challenges, including excessive workloads, high pressure, and burnout (Doherty, 2020; Madigan and Kim, 2021). These issues contribute to high rates of teacher turnover in the education sector and widespread job dissatisfaction among educators. Teacher turnover is a costly issue, impacting not only government finances but also the allocation of educational resources. In recent years, the exodus of experienced teachers from the classroom has garnered increased research focus, particularly concerning the factors influencing teacher retention (Mason and Matas, 2015; Rinke and Mawhinney, 2017) and the early career development of educators (Trent, 2019; Manuel, 2003; Bell, 2021; Bryson, 2021). These studies aim to enhance teacher education programs and foster a workforce of more dedicated and effective educators.

The factors that influence a teacher’s decision to remain or leave the profession are complex and multifaceted. The study by Mason and Matas (2015) identified internal factors such as commitment, conceptualized as positive psychological capital, as being instrumental in these decisions. A teacher’s commitment, viewed as an insight into the educators’ internal experiences, can be developed into psychological capital, which enables them to overcome the challenges associated with teaching. Other studies (Kyriacou et al., 2003; Hong et al., 2018; Wang et al., 2023) found that the motivations behind choosing a teaching career are crucial in shaping and sustaining teaching commitment, with various motivations correlating with different levels of commitment. Moreover, Schonert-Reichl (2015) demonstrates that teachers’ deep-seated dedication and commitment to education can be bolstered during their pre-service training through specialized professional education. Such findings underscore the importance of addressing the motivations and psychological resources of teachers to foster a positive perception of teaching during their early career stages.

Pre-service teachers are defined as ‘student teachers who are enrolled in a teacher education program and working toward teacher certification’ (Keengwe, 2022, p. 15). Understanding the motivations behind pre-service teachers’ career choices and identifying the elements that nurture such motivations are beneficial for enhancing in-service teachers’ dedication and reducing turnover rates over the long term. There are myriad reasons why pre-service teachers may select teaching as their lifelong profession. The reasons range from external motivations, such as the prospect of a stable income, to internal drivers, such as the fulfillment of self-actualization. Some studies (Kyriacou et al., 2003; Hong et al., 2018) have found a strong correlation between high levels of commitment and intrinsic or altruistic motives for choosing a teaching career. Conversely, those with a lower level of commitment often cite extrinsic motivations as their impetus for entering the profession. One significant factor influencing the desire to teach is altruistic motives (Chan, 2006; Tekin, 2016; Aydemir and Er, 2023), such as enjoying the company of children or enjoying prosocial behavior. Accordingly, this study focuses on examining the altruistic motivations influencing pre-service teachers’ career decisions.

## 2 Literature review

### 2.1 Significance of pre-service teachers’ altruistic motivation In career choice

Examining the altruistic aspects of pre-service teachers’ motivation is critical not only because of practical concerns such as teacher turnover but also because of its potential to increase individual job satisfaction and expectancy. For instance, An et al. (2021) demonstrated in a study focused on the Chinese educational system that motivational factors account for 67% of the variance in pre-service teachers’ satisfaction with their career choice, particularly in relation to intrinsic career values such as social utility. Additional studies (Kyriacou et al., 2003; Amzat et al., 2022; Wang et al., 2023) have indicated that despite negative aspects such as unsatisfactory salaries, heavy workloads, and lower social status potentially leading to job dissatisfaction, the teaching profession continues to be viewed as valuable and honorable for its social utility and altruistic values. Through the literature above, altruistic motivation in teachers’ careers does prove its unique significance in strengthening teachers’ commitment to teaching careers to improve the situation of teachers’ attrition on a social scale and to intensify the inner satisfaction as a teacher on an individual scale.

Altruistic motivation in choosing teaching as a career presents its necessity and importance when dealing with teachers’ attrition issues and fostering teachers’ positive perception of career satisfaction. Achieving a better understanding of altruistic motivation in pre-service teachers’ career choices would not only help increase the retention of future teachers but also provide an innovative angle in teachers’ professional education to nourish teachers’ altruistic content in their education career motivation. To investigate and promote pre-service teachers’ altruistic motivation, it is vital to provide a clear definition of altruistic motivation in the context of pre-service teachers’ career choices.

### 2.2 Definition of pre-service teachers’ altruistic motivation

Over the past century, the concept of altruism has been a subject of interest among educational psychology scholars, particularly in the context of pre-service teacher development. Although discussed within various theoretical frameworks, altruistic motivation has rarely been the focal point as an independent element. Mori (1965) was one of the first to identify factors that contribute to the choice of teaching as a career, with social, interpersonal, and ethical reasons being indicative of altruistic motivations. In the early 1990s, some scholars mentioned that altruistic goals should be regarded as one of the primary reasons for choosing teaching as a career (Brookhart and Freeman, 1992). Another scholar (Yong, 1995) conducted a study of gathering components, such as working with children and contribution to society/country, as altruistic motivation. Later on, a three-factor motivation theory, extrinsic (such as stable income), intrinsic (such as feeling intellectually stimulated), and altruistic motivations (such as wanting to make a difference in society) (Rutten and Badiali, 2020; Wang and Wang, 2023), was gradually acknowledged and the concept altruistic motivation presenting the altruistic elements in pre-service teachers career choice started to prevail in the academic field. Another model, Factors Influencing Teaching Choice (FIT-Choice) (Watt and Richardson, 2007), focuses on specific values and expectancies, which includes social utility value being regarded as content of altruistic motivation.

It is clear that the definition of altruistic motivation still has not reached a consensus. Former definitions are variants. Friedman (2016) put forward that ‘teachers’ expectations for self-expression through teaching are directed toward the fulfillment of altruistic needs (instilling knowledge, caring, bestowing friendship)’ (p.1). Heinz (2015) suggested that ‘Individuals entering teaching for altruistic reasons see teaching as a socially worthwhile and important job; they want to contribute to society and work with/help children and adolescents.’ (p.9). Many researchers prefer to list out what altruistic motivation contains as the definition of altruistic motivation. Exemplary lists or phrases include ‘service to others’, ‘the desire to help and support students’, ‘make a difference’, ‘contribute to society’, and ‘answer a calling’ (Fray and Gore, 2018). Other codes include ‘love of children’, ‘to fulfil a mission’, and ‘exhibiting interest in the other’ (Low et al., 2011; Friedman, 2016). Such a method of listing would bring problems such as blurring the distinguishment between intrinsic and altruistic motivations. For instance, the desire to work with children can be nominated as both intrinsic and altruistic motivations (Watt et al., 2012), which demonstrates that using certain phrases to define altruistic motivation could be confusing and superfluous. Even if Watt et al. (2012) realized the need to provide a unanimous scale in FIT, the detailed description of each utility value still used the old-fashioned way by using phrases to define altruistic motivation. Based on the definition problem above, refining the definition of altruistic motivation in pre-service teachers’ career choice is an *a priori* task before investigating its formation and development.

The current study will be first based on the definition Rutten and Badiali (2020) put forward to distinguish the difference between intrinsic and altruistic motivations as they perceive intrinsic motivation relates to teaching experience and altruistic motivation relates to a worthwhile pursuit that can make a difference for children or society. To penetrate into the altruistic content, this study summatively composed the altruistic motivation definition inspired by Rutten and Badiali (2020) to adopt a goal-oriented definition and included a wider object of altruistic content (family or specific individual that teachers try to benefit). This study defines altruistic motivations as motivation relating to viewing teaching as a worthwhile pursuit for its benefit to the society (macro scale), the younger generations (medium scale), and the people in teachers’ daily realistic environment (micro scale).

### 2.3 Factors affecting pre-service teachers’ altruistic motivation

This study endeavors to elucidate what shapes and fosters altruistic motivation. The existing literature is fragmented, lacking a cohesive structural or theoretical framework. Research by Chang et al. (2022) revealed a connection between social relationships and the motivation of students and teachers, suggesting that teachers’ social attitudes contribute to varying degrees of job satisfaction and influence their willingness to teach. The moral dimensions of teaching, as explored by Osguthorpe and Sanger (2013), stressed the importance of moral purposes in teachers’ social perceptions of their profession. Social-Cognitive Career Theory, proposed by Lent et al. (1993), offers a structured approach, positing that self-efficacy serves a mediating function in career selection. Moreover, a study contextualized within the Chinese education system (Wang and Gao, 2013) advocates for the integration of social issues such as equity and justice into teacher training programs. Being illuminated by studies about people’s social-cognition, teachers’ cognition toward social topics and social wellbeing would be one salient research direction behind pre-service teachers’ altruistic motivation. Taking teaching as a career that benefits the world and provides insight into social topics could be the premise for altruistic motivation for pre-service teachers.

While teaching is often viewed as a profession demanding substantial rational intelligence, emotional factors are also recognized as influential in career choice. Research has demonstrated a positive association between various emotions and the motivation to teach. Feelings of joy in working with students, affection for children, emotional connections, a sense of identification with the profession, and a commitment to the educational vocation and students have been commonly cited by teachers (Kyriacou et al., 2003; McIntyre, 2010). Positive emotional feedback from teaching activities enhances teacher confidence and fosters a lasting interest in the profession (Wang and Wang, 2023). Furthermore, empathy, which often arises from witnessing the negative experiences of others, has been extensively studied for its impact on career motivation. An early study by Aronfreed and Paskal (1965) suggests that empathy may evolve into altruistic motivation. Some studies also focus on the lay-behind correlation between empathy work and altruism (Van Lange, 2008; Li et al., 2022). In addition to empathy, negative emotions and experiences also need to be considered as a reinforcement of altruistic behaviors (Cialdini and Trost, 1998; Blair and Mitchell, 2009; Thielmann et al., 2020). Cialdini et al. (1981) put forward a three-step model to demonstrate how the sad emotions affect prosocial behavior, which suggests that altruism is capable of transferring bad mood into a treat for people. Negative emotions could trigger prosocial behavior, but to know exactly how the process goes still requires further understanding. Introducing emotion as one of the vital elements in investigating the pre-service teachers’ altruistic motivation is significant as it imposes a strong dynamic on choosing to teach for altruistic outcomes.

In the current study carried out in the Chinese context, some of the realistic elements are to be considered. The realistic element concerns pre-service teachers as individuals in different contexts or living environments to obtain altruistic motivation. The family reason is rather powerful in Chinese culture, affecting the younger generation’s career choices. Many undergraduates choose a certain job out of family expectancy from the previous generation or family responsibility for the next generation. Sometimes, family culture or family tradition impacts undergraduates’ career choices when it comes to the family’s opinion that teaching is a novel and stable career (Li, 2021; Zhang, 2022), which can be viewed as a kind of altruistic motivation to satisfy certain family needs. Another realistic theme would be occupational preferences based on various personal traits (Franz et al., 2022). Occupational preferences, under the influence of one’s personality or self-perception (Watt and Richardson, 2007), might be a vital element for inclining to certain feature careers. Teaching has the feature of helping others and fulfilling one’s values. This is another indispensable realistic element that should be taken into consideration when understanding how real situations work on altruistic motivation among pre-service teachers. Realistic factors concerning people around pre-service teachers and teachers’ own occupational preference for altruistic features enable innovatively digging into the formation and development of altruistic motivation in pre-service teachers’ career choices. Yet, to achieve this purpose, some theoretical obstacles still exist.

Despite the three-component model put forward by Atabaeva (2019) being well-structured and clearly classified in an individual dimension, it has noticeable deficiencies. First of all, this component-based model makes it easy to confuse whether it is talking about what influences altruistic motivation or the content of motivation itself. Second, factors within the component seem to be blurred and overlapping. For instance, some emotions are sometimes hard to define, whether they are caused by others or themselves, such as empathy. The motivational component overlaps with altruistic motivation. Meanwhile, motivational components involve personal traits and some egoistic factors. The former one may need to be better categorized, and the latter one is still an academic conflict about whether altruistic behaviors out of egoistic reasons can be regarded as altruism or not. Finally, the original model needs to be modified to study pre-service teachers’ altruism in a particular cultural context. To sum up, when trying to seek the mechanic process or elements behind altruistic motivation, existent theory and discussion seem to be in fragments, so a more structural and complete theoretical framework is desired. To overcome previous model deficiencies and advance beyond the existing models, a new three-element model with a new typology of each element is proposed in the current study.

### 2.4 Theoretical framework

**Figure 1:**
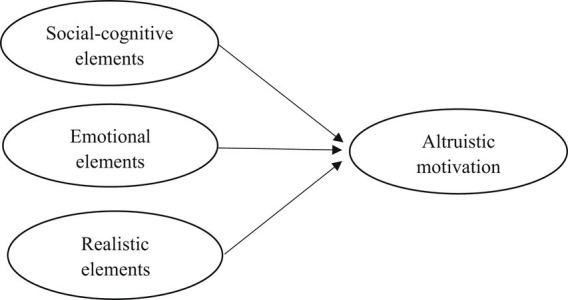
Theoretical framework of this study.

To advance beyond previous models (Cialdini et al., 1981; Atabaeva, 2019) in examining the genesis and evolution of altruistic motivation, particularly in the context of pre-service teachers’ career decision-making in China, this study introduces a tripartite theoretical framework (Figure 1). The three determinants of altruistic motivation delineated within this framework are as follows:

1. Social-cognitive element, which refers to cognitive factors related to social topics.
2. Emotional element, which is defined as teachers’ positive emotion and negative emotion.
3. Realistic element, which refers to teachers’ family reasons and personal occupational preference for choosing teaching as a career.

The advancement and innovation of this newly proposed framework excels in three aspects. First, it is based on but goes beyond old models by clearly describing three factors that influence the development of altruistic motivation, which is different than the confusion or combination of components in altruistic motivation or factors that influence the development of altruistic motivation. Second, this proposed framework has three distinct yet logically connected structures to guide the study and the findings, which goes beyond previous literature that lacked a synthesized and holistic framework to organize the research findings. Third, this theoretical framework considers the role of culture in the development of altruistic motivation and is open to include other culture-adaptable elements for future studies.

## 3 This study

To further investigate the formation and development of altruistic motivation in pre-service teachers’ career choices, we conducted the study based on the three-element theoretical framework and aimed to answer the central research question:

- What are the general profiles of pre-service teachers’ altruistic motivation?
- What are the influencing factors to cultivate pre-service teachers’ altruistic motivation in choosing teaching as a career?

## 4 Method

### 4.1 Participants

According to the Ministry of Education (2023) report, there are currently over 0.6 million students enrolled in teacher education programs in each academic year (years 1–4), i.e., over 2.4 million pre-service teachers. Among all the universities that have registered teacher education programs, there are six top normal universities that set up the Free Teacher Education (FTE) program that takes 12,000 students each year. FTE program supports students to study teacher education program free of tuition but also requires students to compulsorily undertake teacher jobs for at least 5 years after graduation. In other words, pre-service teachers who choose the FTE program will surely become teachers in the near future.

A stratified sampling method was utilized to recruit 18 participants from the top normal university with a Chinese Free Teacher Education (FTE) program.

Given that our research topic is to explore how participants’ altruistic motivation is developed, we need to select potential participants who possess the features of altruistic motivation. Therefore, according to the definition of altruistic motivation, we developed a short survey to measure the level of altruistic motivation and spread it among pre-service teachers by convenience sampling. In total, we collected 123 pre-service teachers’ survey responses and screened out 51 who showed a high level of altruistic motivation. To balance between eventual participants’ demographic backgrounds, such as gender, year, and major, to select as many representative samples as possible, we authors picked out a pool of 25 backup participants to reach out for their consent and time for a one-to-one interview. Eventually, 18 pre-service teachers agreed to be interviewed. As illustrated in Table 1, a balanced representation from the entire pool of surveyed students, the selected stratified sample comprised 9 male and 9 female participants. The academic year distribution included 5 freshmen, 5 sophomores, 4 juniors, 2 seniors, and 2 postgraduate students. Regarding academic disciplines, 8 hailed from the humanities (Chinese literature and English) and social sciences (Politics and History), 9 from STEM fields (Geography, Chemistry, Physics, and Biology), and 1 specialized in early childhood education. For the purpose of anonymity and ease of reference during the analysis, participants were coded from S1 to S18 according to the sequence of their interviews.

**Table 1:** Demographic information of sampled participants.

Label	Grade	Gender	Major
S1	Year 3	Female	Chinese literature
S2	Year 3	Female	Chinese literature
S3	Year 3	Female	Early child education
S4	Year 2	Male	English
S5	Year 2	Female	Math
S6	Year 1	Male	History
S7	Year 1	Male	Chinese literature
S8	Year 2	Male	Math
S9	Year 1	Female	Math
S10	Year 1	Female	Biology
S11	Year 1	Male	Geography
S12	Year 2	Female	Politics
S13	Year 3	Male	Math
S14	Year 4	Male	Math
S15	Year 4	Female	History
S16	Year 2	Male	Politics
S17	Postgraduate 1	Male	Math
S18	Postgraduate 1	Female	Chinese literature

### 4.2 Data collection

Each participant provided written consent before participating in the study. Subsequently, 18 sets of 30–40 min semi-structured one-to-one individual interviews were carried out, and the audio was duly recorded. The interview protocol encompassed seven main interview questions (Appendix 1), which were devised in accordance with the three-element theoretical framework guiding the study. Following the interviews, the audio recordings were meticulously transcribed. These transcriptions were then thoroughly cross-checked for accuracy against the original audio records. All interviews were initially conducted in Chinese; subsequently, the transcripts were translated into English for analysis.

### 4.3 Data analysis

For the purpose of identifying and interpreting patterns within the interview data, thematic analysis was employed. The process followed the structured guidelines for thematic coding set out by Braun and Clarke (2006) and Bryman (2012). The analysis was systematically organized around the three themes that constitute the proposed theoretical framework: (1) social-cognitive element, (2) emotional element, and (3) realistic element. Codes pertinent to the social-cognitive theme were created to demonstrate the impact of participants’ understanding of social issues on their altruistic career motivations. Emotional theme codes were grouped to depict how participants’ past emotions contributed to the development of altruistic motivation. Finally, codes related to the realistic theme were used to highlight how the individual’s environment and personal characteristics influenced the development of altruistic motivations toward a teaching career.

## 5 Findings

Based upon thematic data analysis, the research findings will be unfolded by the newly constructed three-element model that delineates the formation and development of pre-service teachers’ altruistic motivation in choosing teaching as a career, i.e., social-cognitive component, emotional component, and realistic element.

### 5.1 General profiles of pre-service teachers’ altruistic motivation

According to the previous literature review and current research, on a macro scale, altruistic motivations in teacher candidates’ career choice regarding teaching as a worthy occupation are its potential to make a difference to society, even the entire mankind in the educational field. On a medium scale, it is such motivation or affection to support the development of younger generations in their educational progress. On a micro scale, to benefit the people in pre-teachers’ daily lives (such as their parents or close friends) or live up to their expectations can also be interpreted as altruistic motivations in pre-service teachers’ career choices.

### 5.2 Social-cognitive element for altruistic motivation in choosing teacher as a career

Social-cognitive elements could be defined as cognitive factors related to social topics that lead to altruistic motivation. According to the interview data, participants’ social-cognition for altruistic motivation was cultivated by initiative and passive approaches. The initiative approach stands for participants to start the cognitive process of their own free will. The passive approach means participants start their cognitive process through external factors, such as education of knowledge or others’ (such as friends’ or relatives’) persuasion.

Social-cognitive elements would extract two cognitive contents from the data analysis (Table 2): (1) participants’ observation on the uneven allocation of educational resources and other social topics, such as the deficiency of examination-oriented education and social stereotypes; and (2) participants’ reflection on practical issues in education.

**Table 2:** Social-cognitive element.

Social-cognitive element	Categories	Number of codes
1. Social observation and cognition	1.1 uneven allocation of educational resources	23
	1.2 deficiency of examination-oriented education	14
	1.3 social stereotype	9
2. Reflection and plan	2.1 reflection on practical issues in education	19

In terms of uneven allocation of educational resources, it refers to the phenomenon that the distribution of educational resources is so unbalanced between financially rich or poor areas and between academically “good or bad” students. Such an imbalance in students’ availability to learning hardware and software stimulated pre-service teachers’ motivation to change such social phenomena in education. On a macro scale, resources are usually converged in developed regions (*n* = 8). On a micro scale, students with better academic achievements usually received more attention and care in class (*n* = 4).

Eight participants mentioned the situation happened on a macro scale, with educational resources in different regions. Taking S6’s answer as an example:

*I went to Guangdong province with my parents before I went back to Guangxi province to prepare for college entrance examination. During the one-year preparation, I realized there is a huge disparity between Guangdong and Guangxi province in educational resources, for Guangdong is a more developed province. Moreover, I think such uneven allocation is not fair for less developed region students and I should do something to help poor area students have richer education resources.*

From S6’s description, his own study experience indicated his awareness of the problem. His cognition came from his witnesses of the contrast in different regions. Although he did not believe a single person would be able to change the situation on a large scale, his way to form a contribution was to serve as a qualified teacher to disseminate advanced ideas and skills in learning to the regions in need. This social-cognitive factor then drove him to choose teaching as his career choice to fulfill his thought, considered to be his altruistic motivation.

Other four participants mentioned educational resources problems on a micro scale, as S12 did:

*Teachers tended to pay more attention and show more interest in teaching to those students with more academic achievements or better potential in achieving higher scores. However, when it comes to students without such achievements or ability, teachers are sometimes lack of awareness to provide guidance in both daily life and study progress.*

S12 brought a personal insight into the often-overlooked needs of students who may not be top academic performers. She emphasized the psychological and educational neglect these students face, which fosters a negative cycle of disengagement and declining mental wellness. Through her increased understanding of these issues, S12 felt compelled to pursue teaching with a specific aim: to be an inclusive educator who supports every student’s learning and emotional wellbeing. The drive to make a difference in the lives of all students, not just the academically gifted, is a clear manifestation of her altruistic motivation for entering the teaching profession.

In addition, social topics such as the deficiency of examination-oriented education and social stereotypes also occurred to the minority of participants (*n* = 5). It is obvious that the observation of the phenomena closely bonded with pre-service teachers’ altruistic motivation in their career choices. Drawing from the interview data, it is clear that the observation of uneven allocation of educational resources is a boost to pre-service teachers’ altruistic motivation.

In terms of reflection on practical issues in education, it refers to participants not only seeing or becoming aware of the social topics but also proposing or putting out their own thinking and plausible solutions to tackle practical issues, such as putting forward a reasonable or feasible idea to facilitate modern education in China. S5 would be an applicable example as he continues to reflect on the topic of uneven allocation of educational resources.

*I had a physics professor using internet as a tool to efficiently convey physical knowledge and vividly simulated experiment. It would be very ideal for me to become a teacher using internet as a method to deal with deficiency in uneven allocation in educational resources.*

In S5’s answer, he presented a picture that online educational resources could make up for the current deficiency in educational resource allocation, through which he came across the idea of becoming an online education teacher as his future career. He wishes to use the internet to pass on advanced learning knowledge to those disadvantaged regions. His reflection presented a further social-cognitive element that provided inspiration when encountering obstacles related to social topics, which undoubtedly contributed to the altruistic motivation for pre-service teachers.

In the interpretation of the social-cognitive element, uneven allocation of educational resources on both macro and micro scales would be the cognitive source to cultivate participants’ altruistic motivation. Not only pre-service teachers’ observation but also their further reflection on the practical educational issues indeed formed their altruistic motivation.

### 5.3 Emotional element for altruistic motivation in choosing teacher as a career

Emotional elements facilitate pre-service teachers’ altruistic motivation in choosing teaching as a career in two aspects (Table 3): positive emotion and negative emotion.

**Table 3:** Emotional element.

Emotional element	Categories	Number of codes
1. Positive emotion	1.1 positive affection towards teachers	21
	1.2 self-identity of becoming a teacher	16
2. Negative emotion	2.1 dissatisfaction or criticism of bad teaching	15
	2.2 empathy for students in need	11

Positive emotions, such as affection, self-accomplishment, and confidence, have substantially affected sampled pre-service teachers’ altruistic motivation. Almost every participant (*n* = 17) mentioned that positive emotional experiences serve as a significant factor, but this aspect can still be divided into two facets: (1) affection toward teachers (*n* = 15) and (2) self-identity of becoming a teacher (*n* = 8).

First, to illustrate the affection toward teachers and how such emotion affects pre-service teachers, it would be evident to see its impact from what S6 and S15 said.

S6: *When I was a kid in primary school, I considered myself as kind of problem student and was taken as negative example in class and I never thought to change my attitude towards life and study before I met the teacher that changed my life. Her soft words and sincere caring for me made me a brand-new person.*

S15: *Since my grandparents and some of my relatives are teachers, I can easily feel how knowledgeable they are, and how devoted they are to their students. Teachers as a ideal career since then always appeals to me.*

Participants like S6 and S15 showed different kinds of affection to the teacher. Participant S6’s experience can be interpreted as affection to a specific teacher; meanwhile, S15 showed affection to the group of teachers or teaching as a specific career. For S6, his gratitude for his teacher drove him to take his teacher as a role model and go after his teacher’s path in making positive changes for his future students. For S15, under the influence of the teachers in her family, she cultivated a strong sense of identity in this career and hopefully to become a teacher like them to dedicate herself to the education of the next generations. Their resemblance represented the role of positive emotion in cultivating their altruistic motivation by providing exemplary models and career identity.

Whether such affection is to a specific teacher or the teachers as a whole, participants shared the same positive emotion toward teachers, which fosters pre-service teachers to follow the path of exemplary teachers.

Second, other than emotion toward others, positive emotion toward oneself would also promote altruistic motivation. S5’s story tells how his altruistic motivation is affected by self-identity.

S5: *When we were in junior high school, our teacher encouraged us students to lecture our classmates. I enjoyed this satisfactory feeling and sense of achievement when I gave lectures to my classmates because I taught others to solve the problems successfully.*

S5’s account offers insight into the role of self-identity. This concept relates to recognizing one’s necessity and the accomplishment felt when one’s contributions are valued—altruistic actions often validate and uncover personal worth. Motivated by this affirmation and eager to perpetuate such rewarding experiences, S5 was driven to pursue teaching, aspiring to support students who struggle academically. The realization of self-worth through a vocation characterized by altruism, such as teaching, is a form of positive emotion that can inspire a teacher’s altruistic inclination. Overall, the positive feelings toward the profession and the individual’s sense of self-identity emerge as influential factors in nurturing pre-service teachers’ altruistic motivation.

Negative emotions, such as dissatisfaction, distaste, or empathy, toward all sorts of educational phenomena are considered to be another emotional factor. Six participants mentioned dissatisfaction or criticism of bad teaching and three mentioned empathy for students in need. S8’s response shows how his dissatisfaction with his teachers’ teaching style contributed to his altruistic motivation.

*A high school teacher used to say “You are a terrible learner. Why do not you give it up earlier and to be a waiter in the restaurant?.” This teacher’s words really made me feel sick and hurt.*

S8’s account presents a poignant example of the impact that teachers can have when they neglect their students’ emotions. His own adverse experiences, characterized by feeling undervalued and dissatisfied with his teachers’ pedagogical approaches, fostered a resolve to eschew such detrimental teaching practices. Ignited by such negative emotion, to boycott this kind of improper teaching method that can cause unnecessary emotional damage to children, S8 wanted to be a caring and encouraging teacher in the future. S8’s data showed how a negative attitude can result in altruistic motivation by boosting the intent to resist bad teaching.

Regarding empathy, it often stems from recognizing and relating to the struggles faced by students within the educational system. As evidenced by S12 and S3, this understanding prompts a compassionate response, fueling the desire to aid those who are facing these challenges.

S3: *I lived in the countryside when I was younger. I know that many of my classmates had to give up studying and chose to become housewives due to family reasons. I felt empathy with those classmates.*

S12: *In fact, I actually just returned from teaching in Guizhou last month, kids living there with poor family condition not only have to deal with the problem of study, but also have to worry about how to make a living to support their family. Nonetheless, their passion to learn knowledge touched my heart.*

Having witnessed the struggle of students in undeveloped regions, S3 and S12 attach their concern and empathy to those students in need. S3 feels worried about her friends since she knows exactly what it is like to live in a village where the importance of education is usually underestimated. She emphasized that changing her friends’ next generations’ destinies became the strongest engine for her to teach. In other words, S3’s altruistic motivation came from her empathy with her friends. As for S12, by feeling compassion for helping kids, she set an aim to become a teacher. For both S3 and S12, empathy is not just an emotion but a transformative force that propels them toward a teaching career, reinforcing their altruistic intentions.

In conclusion, emotional influences on career decisions play a crucial role. Both positive emotions, such as affection for the profession and a sense of personal achievement, as well as negative emotions, including dissatisfaction with past educational experiences and empathy for disadvantaged students, significantly contribute to shaping pre-service teachers’ motivations for entering the field of teaching.

### 5.4 Realistic element for altruistic motivation in choosing teacher as a career

The realistic element (Table 4) influences the formation and development of altruistic motivation through family-related factors (*n* = 7), such as parental expectations, family tradition, and aspirations for the next generation’s education, as well as personal occupational preferences (*n* = 5).

**Table 4:** Realistic element.

Realistic element	Categories	Number of codes
1 family influences	1.1 parental expectations	14
	1.2 family tradition	4
	1.3 aspirations for the next generation’s education	6
2 occupational preference	2.1 altruistic nature of educational careers	8
	2.2 realization of social value	9

Regarding family influences, S13 indicated that his decision to pursue a career in teaching was motivated by a desire to fulfill his family’s expectations.

S13: *Being a teacher means a more stable job with stable income to live up for my parents’ expectation and better educational resources that could provide to my future generation.*

Motivated by his parents’ wishes and the wellbeing of his future family, S13’s decision to become a teacher was significantly influenced by familial considerations. In other words, the influence of family factors could gradually lead to altruistic motivation for pre-service teachers.

Regarding personal occupational preferences, a subset of participants (n = 5) expressed a strong affinity for the altruistic nature of educational careers.

S8: *My job preference is that I can create value by my hands not only to myself but also to benefit other people. Teaching then certainly becomes my ideal career. Personally, to become a teacher can cultivate my self-realization of helping others.*

S8 articulates that the appeal of teaching lies in its capacity to enable the realization of social value. For him, the teaching profession is an ideal platform to actualize this value. Given that altruistic outcomes are the objective of such occupational preferences, it is evident that the vocational inclinations of pre-service teachers can nurture their altruistic motivations in choosing their careers.

Apart from social-cognitive and emotional elements, both family factors and personal occupational preference are reflected in realistic terms in the process of forming altruistic motivation in pre-service teachers. The realistic element particularly reflects local Chinese collectivist culture (Duan, 2021), in which individuals’ decisions and behaviors are highly impacted by subjective norms, such as family members, relatives, friends, etc. Real-world situations, such as pre-service teachers’ surroundings and occupational preferences, would also need to be taken into account for developing altruistic motivation.

## 6 Discussion

### 6.1 The three-element model in cultivating pre-service teachers’ altruistic motivation

Key and up-to-date research trends in motivation are basically unfolding in two tracks: one is content-oriented, which tells what motivation is, and the other is process-oriented, which explains how motivation occurs (Bright et al., 2019). This study focuses on constructing the process of motivation, specifically on pre-service teachers’ altruistic motivation in teaching.

The social-cognitive element shows observation and reflection on social topics in education associated with pre-service teachers’ altruistic motivation in their teaching career, which emphasizes the social-oriented traits of social wellbeing. By contrast, previous studies on social-cognitive tended to relate to self-development and emphasized social modeling, self-regulation, and self-efficacy (Lent et al., 1993; Bandura, 2012; Bembenutty et al., 2016), which could not represent a full picture of social-cognition. The present study corroborates the presence of the social-cognitive dimension in the emergence of altruistic motivation. Taking social inequity in education, for instance, after noticing such a social phenomenon, some participants tend to ask themselves what they can do about the situation to better distribute educational resources. Actions such as feeding back to disadvantaged regions themselves or becoming an online teacher to bring advanced resources to the needy stimulated their altruistic motivation when they chose teaching as a career. The finding in the Chinese context also echoes the study carried out in the Chinese FTE program (Wang and Gao, 2013), suggesting that social topics-related education should be included in teacher candidates’ curriculum. Within all the topics participants have mentioned, uneven allocation of educational resources on both macro and micro scales is most frequently discussed, indicating that insight into social educational issues is essential to discuss in the teacher candidate program. Professional training for pre-service teachers can enhance teachers’ altruistic motivation by cultivating their social-cognition.

The emotional dimension constitutes a significant facet intricately linked with altruistic motivation. Positive emotion in cultivating altruistic motivation in pre-service teachers occurs before stepping into the teaching field. Examples such as gaining self-identity in teaching when receiving acknowledgment from teaching peers or affection from their former teachers are frequently mentioned. Such positive emotion remains a booster of pre-service teachers’ altruistic motivation. The mechanic lying behind can be interpreted as a positive vibe from the altruistic aspect of an education career, which can reinforce the will in teacher candidates to become a teacher for social wellbeing. On the other hand, negative emotions, including empathy, also play an important role in pre-service teachers’ motivation, which echoes previous literature emphasizing the possibility of turning bad emotions into psychological capital and then turning into prosocial behaviors (Cialdini and Trost, 1998; Blair and Mitchell, 2009; Thielmann et al., 2020). From the interview data, negative emotions from previous experiences are related to two specific groups: unqualified teachers and disadvantaged students. To show a teaching model to benefit future students and to help those disadvantaged students are mostly established as altruistic motivations. It is worth noticing that pre-service teachers who used to be disadvantaged students stood as an important group for future academic research. They tend to have strong altruistic motivation under the enhancement of two kinds of negative emotions: dissatisfaction toward teachers and empathy with their disadvantaged peers. Among the two kinds of negative emotions, empathy is the most frequently mentioned emotion element. Whether one has been in a disadvantaged position in person or knows the poor situation others are in, empathy is the primary emotion that triggers altruistic motivation. As a result, it becomes a valuable psychological resource for teachers to gain their own altruistic goals in education.

In addition to social-cognitive and emotional elements, the realistic element is an innovative element in the current study. Drawing on the insights from previous studies that focused on individual perspectives (Watt and Richardson, 2007; Heinz, 2015; Atabaeva, 2019), the realistic element is conceptualized to accentuate the characteristics of individuals and their environments, particularly within the Chinese context. In China, family considerations are often paramount. The pursuit of a stable career to address parental expectations or ensure enhanced opportunities and educational resources for descendants is recognized as a significant driver of altruistic motivation. Thus, altruistic motivation comes from realistic elements, such as whose families think highly of teaching or with promising prospects for the next generations. Occupational preference is the other realistic element in pre-service teachers’ career choices. According to the interviewees, altruistic motivation is formed among those who are attracted by altruistic traits in their educational careers. The attraction to teaching also relates to the Chinese cultural context. Pre-service teachers’ occupational preference is affected not only by their individual inclination but also by regarding teaching as a family career (Hong et al., 2018; Duan, 2021). The realistic element should become an indispensable element to dig into for its explanatory power in clarifying the formation and development of altruistic motivation.

The current study primarily constructs the three-element framework to illustrate the formation and development of altruistic motivations in pre-service teachers’ career choices as one theoretical contribution. On the one hand, this study notices the vacancy in investigating the process-motivation theory when it comes to pre-service teachers’ altruistic motivations, as this theoretical framework enables the research to investigate the origins and developments of altruistic motivations in a more structural way.

### 6.2 The relationship among three elements in cultivating pre-service teachers’ altruistic motivation

Furthermore, a significant finding in the current study is identifying the possible relation among three elements, which is an important facet that lacks further discussion in previous studies.

Modern neuroscience has proven that cognition and emotion are closely related to human brain processing (Phelps, 2006; Pessoa, 2008). It is found that social-cognitive and emotional elements have mutual effects. Participant S8 mentioned that after realizing the uneven allocation of educational resources from social media, he developed strong empathy with students in disadvantaged positions, and this emotion drove him further in his career choice. Realization and reflection on the social topics on a cognitive basis would trigger emotions that further promote pre-service altruistic motivation to make a difference and achieve altruistic goals. Simultaneously, the emotional element contributes to the social-cognitive process. Participant S15 said that her dissatisfaction and sickness of Chinese examination-oriented education (Zhang et al., 2021) in the first place encouraged her to reflect on the current situation and come up with some possible solutions, such as developing a new evaluation system for the teaching subject. It is obvious that the social-cognitive element and emotional elements are intensively bonded in cultivating pre-service teachers’ altruistic motivation.

The other noticeable relation is that some participants tend to associate their certain social-cognitive or emotional elements with realistic elements. On the one hand, the realistic element could be a reinforcement of the social-cognitive element. S7 mentioned that his consensus with his parents’ expectancy to help with education in their less advanced hometown is a booster of his altruistic motivation in his career choice. Parents’ expectancy (Heinz, 2015; Hong et al., 2018; Duan, 2021) as realistic reasons contribute to his attention to the local educational dilemma, eventually leading to his altruistic tendency in career choice. On the other hand, emotional elements can influence realistic elements such as occupation preference. S15 said that her affection for a teaching career came from her grandparents and relatives who are teachers. Their altruistic working attitude was something she admired and would love to inherit. Her positive emotion toward teaching formed her occupational preference for a career with altruistic traits. The realistic elements exhibit a mutual relation with other elements in facilitating altruistic motivation, which imposes its important position in future studies.

## 7 Conclusion

This study constructs a structural and functional model based on existing research, combining the three-element model of social-cognition, emotion, and realistic themes, to explore how pre-service teachers’ altruistic motivations developed in a qualitative method. Findings indicate that social-cognitive elements influence altruistic motivation through observation and reflection on social issues or practical educational challenges. Emotional elements are participants’ positive and negative emotions in their education process. Realistic elements include family influences and personal occupational preferences that play a role in the decision-making toward a teaching career. The theory proposed in this study can serve as a theoretical basis for future research on the development of altruistic motivations.

## 8 Limitation and implications

This qualitative study has limitations of a relatively small sample size compared to a quantitative study. Thus, the recommendation is to employ a larger sample size by surveys or other instruments to test the three-element model in a wider population and more research contexts.

In general, the current study makes a theoretical contribution to breaking the vacancy in studies about the formation and development of altruistic motivation in pre-service teachers’ career choices. Meanwhile, this study also provides a feasible model for future investigation to adopt a more structural insight into how altruistic motivation in pre-service teachers is formed. In addition, the relations among the three elements provide a micro aspect for future studies to focus on explaining the mutual influence of the elements for the nuanced development of altruistic motivation.

The practical implication of this study inspires future educational practice. In pre-service professional training programs, findings in the current study suggest that courses related to where teachers’ motivations come from could help pre-service teachers to better understand their teaching motivations and establish their altruistic goal-oriented perceptions for lifelong teaching careers. For policymakers, policies enhancing pre-service teachers’ altruistic motivation would be a significant task for improving teachers’ retention rate and teaching resources in the future.

## Data availability statement

The original contributions presented in the study are included in the article/Supplementary material, further inquiries can be directed to the corresponding author.

## Ethics statement

The studies involving humans were approved by Shenzhen Baoan Academy of Education Science Ethical Committee. The studies were conducted in accordance with the local legislation and institutional requirements. The participants provided their written informed consent to participate in this study.

## Author contributions

ZL: Conceptualization, Data curation, Investigation, Writing – original draft, Formal analysis. WG: Methodology, Supervision, Writing – review & editing, Conceptualization.
