# Supplementary material for: Pre-service teachers’ altruistic motivation for choosing teaching as a career: where does it come from?
Source: Front Psychol. 2024 Apr 2;15:1334470. doi: 10.3389/fpsyg.2024.1334470 (PMC11020075; doi:10.3389/fpsyg.2024.1334470)
Supplement: Supplementary file 1 [file Data_Sheet_1.docx]

**Appendix 1: Interview Protocol**

- Preliminary interview questions

1.Do you have a career plan to become a teacher in the future as you chose the pre-service teacher program?

2.To confirm whether there are altruistic factors in the reasons for choosing the profession.

2.1) What do you think is the most important reason for you to choose teaching as your career?

2.2) Is there one of the following elements in your motivation to become a teacher?

Societal level: e.g. whether you want to become a teacher to contribute to the education of the society, the country, or even the entire mankind.

Individual level: e.g. whether you want to become a teacher because you like to work with the next generation, or because you want the younger generation to have a better future.

3.Introducing the interview theme: this study aims to investigate the factors influencing the altruistic motivation of pre-service teachers in choosing teaching as a career. Through reading the literature and studying theories, we have come to the conclusion that there are three relevant influencing elements: socio-cognitive elements, emotional elements, and realistic elements.

- Main interview questions

1.Socio-cognitive elements

Regarding the socio-cognitive aspect: this aspect refers to the cognitive factors in the human understanding of social issues or social propositions that promote the generation of altruistic motivation. In other words, someone is willing to devote himself to the teaching profession that promotes the fairness of education because of the deep knowledge of the lack of fairness in education; or he wants to become a teacher to make practical contributions to the solution of the problems of exam-based education that appear in the present and be able to make practical contributions to the solution of this problem.

1) Is there any motivating factor of your career plan that is related to the development of education, which is a relatively significant social issue?

2) How did the idea come into your mind? For example, did your parents and teachers tell you to do it or did you get it from your own understanding of the social situation?

3) Can you think of any additional examples in the ream of socio-cognitive elements?

2. Emotional elements

This aspect refers to the fact that people are motivated by some special past experiences or some special emotional factors that promote altruistic motives, for example: you are dissatisfied with your class teacher's education when you were a child, or you are emotionally hurt because of this, which prompts you to want to become a teacher to change the status quo; or out of the love for a former teacher, which makes you want to become a teacher like him. Maybe you want to be like a teacher out of love for a teacher you once had.

1) Was there a "special experience" you had or a "specific emotion" you felt when helping others that influenced you to choose teaching as a career?

2) Can you briefly describe how such feelings or experiences influenced the altruistic component of your motivation for choosing teaching as a career?

3) Can you think of any additional examples in the ream of emotional elements?

3. Realistic elements

For realistic elements, altruistic motivation is based on certain realistic factors or goals. For example, if you choose to become a teacher because you want to fulfill your parents' expectations of you, altruistic motivation is directed at your parents. Another example is to say that someone wants to come back to their hometown and help some of their friends and relatives around them to update their concept of education. It is the main force that acts on certain groups in one's real life environment.

1) Do you think you have any realistic motivation in choosing teaching as a career? If so, can you briefly explain them?

2) Are there any other elements besides the examples you have just mentioned? Or can you think of anything else to add?
